# Results of 3,668 primary total hip replacements

**DOI:** 10.3109/17453674.2012.672095

**Published:** 2012-04-24

**Authors:** BW Schreurs, JWM Gardeniers

**Affiliations:** ^1^Department of Orthopedics 357, Radboud University Nijmegen Medical Centre, Nijmegen, The Netherlands

*Sir*—We have read with great interest the study of the Finnish Arthroplasty Register by Mäkelä et al. (2011) on patients under 55 years who had a primary total hip implant for primary osteoarthritis. This report is a follow-up of the previous report by Eskelinen et al. in Acta Orthop (2005).

We agree with the authors that the population-based outcomes of total hip arthroplasty appeared to be relatively unsatisfactory for younger patients in Finland. However, it is likely that the outcome for young patients with a total hip hip arthroplasty in Finland are even worse than the paper suggests. Unfortunately, and this remains unclear from the abstract, the current study of 3,668 primary hips is based on only 56% of all patients under 55 years who had a total hip implant in the research period in Finland for primary osteoarthritis.

The researchers excluded 2,910 total hip arthroplasties, nearly all noncemented hip, because all noncemented implants with known poor track records as well noncemented implants not fitting in the cementless group 1 (implants with a cementless, straight, proximally circumferentially porous-coated stem and a porous-coated press-fit cup) or 2 (implants with a cementless, anatomic, proximally circumferentially porous-coated stem, with or without hydroxyapatite, and a porous-coated press-fit cup with or without hydroxyapatite) were excluded, as well as those who had been implanted less than 10 times a year. They also excluded cemented total hips inserted with CMW or Boneloc cement, but these numbers are limited we guess. So, even after already excluding the implants with a poor outcome, the results were still somewhat disappointing and no better than cemented hips in the same population group in Finland.

Despite the fact that we are now 5 years after the previous report of the Finnish register on this group of patients, there has been hardly any improvement on the outcome of noncemented hips in young patients. We had in 2005 and 2006 some discussion with the Register on their interpretation based on the study in 2005 suggesting that for younger patients uncemented hips were most attractive (Schreurs et al. 2005, 2006). 5 years later the outcome has not improved and even after a selection with exclosure of the poor performing implants, the conclusions remains the same. Looking at the data with revision for endpoint any reason, modern noncemented hips are in Finland in young patients not superior to cemented hips. Certainly, wear is an important problem and companies have introduced many alternatives during the last years like large metal-on-metal head, newer polyethylene's or ceramic on ceramic. However, one should not be too optimistic that these innovations will solve the wear issue (Sedrakuan et al. 2011).

The outcome of the Finnish register is in line with the conclusions of a literature study we just published on the outcome of total hips in patients under 50 years (de Kam et al. 2011). Although noncemented hips are widely used in patients under 50 years, this trend remains unsupported by survival data. The most reliable results relate to cemented implants. The literature review was done up to 2009-01-01.

Studies from hip registers do have major impact on orthopedic practices, therefore these reports should be of the highest standard with an adequate overview of the literature. The authors suggest that several literature reports based on patients under 55 years do support the use of noncemented total hip arthroplasty and are fulfilling the NICE criteria, meaning a survival of more than 90% at a proven survival of 10 years after implantation with endpoint revision for any reason. However, the cited reports are incorrect. We previously had a discussion on the reports by Kim et al. 2002 and 2003 and McAuley et al. 2004 (Schreurs et al. 2005, 2006). Although these reports are approaching the NICE criterion, as we concluded in our discussion, they still are not updated for unknown reasons. This is remarkable as these reports could have been the first reports worldwide proving that the NICE criteria can be passed by noncemented hips in young patients. The suggestion that Pieringer et al. 2006, Reigstad et al. 2008, Garcia-Rey et al. 2009 and Anseth et al. 2010 are in favor of noncemented hip in patients under 55 years is incorrect. None of these papers is based on young patients under 55 years. The study of Delaunay et al. (2008) is also incorrect, they have a calculated and expected survival at 10 years probably fulfilling the NICE criterion. However, the average follow-up of that series is 7.3 years, and it is known that between 7 and 10 years problems starting to occur.

We are looking forward to the next update on this very interesting study group of the Finnish arthroplasty register and are curious if their findings and conclusions will result in trends to use more proven implants.
