# Supplementary material for: Results of 3,668 primary total hip replacements
Source: Acta Orthop. 2012 Apr 24;83(2):204–5. doi: 10.3109/17453674.2012.672095 (PMC3339538; doi:10.3109/17453674.2012.672095)
Supplement: Related texts

# Letter: Results of 3,668 primary total hip replacements

**Published:** 2012-04-24

**Authors:** K Mäkelä

**Affiliations:** ^1^Department of Orthopaedics and Traumatology, Turku University Hospital, Turku, Finland

*Sir—*We appreciate your interest in our article. Our aim was indeed to study modern cementless implants, not all the failed ones during the evolution of the cementless concept.

It is true that the clinical studies we referred to did not include exclusively patients under 55 years. The patient age in clinical studies we are familiar with is seldom limited to certain age groups. In register-based studies with high amount of patients like ours it is easier to define and analyze specific age groups, like patients under 55 years. In a clinical study from a single unit this is not so easy to do because of the small number of young patients. However, all the studies we referred to included patients under 55 years. We agree that optimally one should probably perform randomized, controlled arthroplasty trials in young patients.

## References

[CIT0001] Anseth SD, Pulido PA, Adelson WS, Patil S, Sandwell JC, Colwell CW (2010). Fifteen-year to twenty-year results of cementless Harris-Galante porous femoral and Harris-Galante porous I and II acetabular components. J Arthroplasty.

[CIT0002] De Kam DC, Busch VJ, Veth RP, Schreurs BW (2011). Total hip arthroplasties in young patients under 50 years: limited evidence for current trends. A descriptive literature review. Hip Int.

[CIT0003] Delaunay CP, Bonnomet F, Clavert P, Laffargue P, Migaud H (2008). THA using metal-on-metal articulation in active patients younger than 50 years. Clin Orthop.

[CIT0004] Eskelinen A, Remes V, Helenius I, Pulkkinen P, Nevalainen J, Paavolainen P (2005). Total hips arthroplasty for primary osteoarthrosis in younger patients in the Finnish arthroplasty register. Acta Orthop.

[CIT0005] García-Rey E, García-Cimbrelo E, Cordero-Ampuero J (2009). Outcome of a hemispherical porous-coated acetabular component with a proximally hydroxyapatite-coated anatomical femoral component: a 12- to 15-year follow-up study. J Bone Joint Surg (Br).

[CIT0006] Kim YH, Kook HK, Kim JS (2002). Total hip replacement with a cementless acetabular component and a cemented femoral component in patients younger than fifty years og age. J Bone Joint Surg (Am).

[CIT0007] Kim YH, Oh SH, Kim JS (2003). Primary total hip arthroplasty with a second generation cementless total hip prostheses in patients younget than fifty years of age. J Bone Joint Surg (Am).

[CIT0008] Mäkelä KT, Eskelinen A, Pulkkinen P, Paavolainen P, Remes V (2011). Results of 3668 primary total hip replacements for primary osteoarthritis in patients under the age of 55 years. Acta Orthop.

[CIT0009] McAuley JP, Szuszczewicz ES, Young A, Engh CA (2004). Total hip arthroplasty in patients under 50 years and younger. Clin Orthop.

[CIT0010] Pieringer H, Auersperg V, Böhler N (2006). Long-term results of the cementless ALLOCLASSIC hip arthroplasty system using a 28-mm ceramic head: with a retrospective comparison to a 32-mm head. J Arthroplasty.

[CIT0011] Reigstad O, Siewers P, Røkkum M, Espehaug B (2008). Excellent long-term survival of an uncemented press-fit stem and screw cup in young patients: follow-up of 75 hips for 15-18 years. Acta Orthop.

[CIT0012] Schreurs BW, Gardeniers JW (2005). Total hip arthroplasty for primary osteoarthroses in younger patients in the Finnish arthroplasty register. Acta Orthop.

[CIT0013] Schreurs BW (2006). Gardeniers JW.Second letter to the editor concerning “Total hip arthroplasty for primary osteoarthrosis in younger patients in the Finnish arthroplasty register” by Eskelinen et al. and correspondence Acta Orthop.

[CIT0014] Sedrakyan A, Normand SL, Dabic S, Jacobs S, Graves S, Marinac-Dabic D (2011). Comparative assessment of implantable hip devices with different bearing surfaces: systematic appraisal of evidence. BMJ.
